# Does Proximal Femoral Morphology Impact Morbidity and Mortality? A Cohort Study of Uncemented Hemiarthroplasties in the Treatment of Femoral Neck Fractures

**DOI:** 10.1016/j.artd.2021.06.010

**Published:** 2021-07-09

**Authors:** Evelyn P. Murphy, Christopher Fenelon, Adrian Cassar-Gheiti, Padhraig O’Loughlin, William Curtin, Colin G. Murphy

**Affiliations:** a Department of Trauma and Orthopaedics, Galway University Hospitals, Saolta Hospital Group, Galway, Ireland; b Department of Science and Engineering, University of Limerick, Limerick, Co, Limerick, Ireland; c Department of Orthopaedics, Cork University Hospital, Cork, Ireland

**Keywords:** Uncemented hemiarthroplasty, Bone cement implantation syndrome, Calcar fracture, Periprosthetic fracture, Hip fracture

## Abstract

**Background:**

To assess outcomes after uncemented hemiarthroplasty stems in the treatment of intracapsular femoral neck fractures over an 11-year period. Mortality rates were assessed, and whether proximal femoral geometry and stem alignment were factors in intraoperative or postoperative periprosthetic fracture (PPF) needs to be identified.

**Materials and Methods:**

A retrospective single-center observational study was conducted of all patients who underwent intracapsular femoral neck fracture treatment using an uncemented prosthesis between January 2008 and December 2018. Primary endpoints included mortality rate, Dorr classification, prosthesis alignment, intraoperative fracture, and reoperation rate for any reason. Subanalysis on collared and uncollared implants was also conducted. Multivariate logistic regression was performed based on Dorr classification for stem alignment, fracture incidence, reoperation rate, implant sizing, and patient mortality.

**Results:**

A total of 536 patients received an uncemented hemiarthroplasty in the study period. The mean patients age was 80.4 years, of which 71% were female. The 30-day mortality rate was 5.2%, with no deaths on day zero or day one. Twenty patients (3.7%) sustained an intraoperative calcar fracture, and 14 patients a PPF (2.6%) at a mean of 1.3 years from surgery. Dorr C type femurs were more likely to develop a PPF (*P* = .001), while valgus stem alignment was associated with PPF (*P* = .049).

**Conclusions:**

This implant has low reoperation rates, low early postoperative mortality, and low 30-day mortality. This large single-center study provides up-to-date information using a contemporary stem in patients with multiple comorbidities. Dorr C femoral morphology and valgus stem malalignment were risk factors for postoperative fractures.

## Introduction

Proximal femoral fractures are increasing in incidence. Considerable debate remains regarding the optimal treatment option for this elderly population. A significant portion of earlier data on uncemented stems did not include hydroxyapatite-coated stems [1,2], and National Institute for Health and Care Excellence guidelines are based on studies that included the use of historic uncemented stems [1,2]. Registry data show comparable survival for ODEP10A stems [3].

There is an increased awareness of bone cement implantation syndrome (BCIS) in the literature [4]. Donaldson et al. defined BCIS as hypoxia, hypotension, or both and/or unexpected loss of consciousness occurring around the time of cementation, prosthesis insertion, reduction of joint, or, occasionally, limb tourniquet deflation in a patient undergoing cemented bone surgery [4]. Olsen et al. studied over 1000 cemented hemiarthroplasties and found an incidence of 28% of BCIS in patients, among whom 1.7% suffered catastrophic events [5]. Although advocates for cemented hemiarthroplasty focus on the decreased intraoperative and postoperative periprosthetic fracture (PPF) risk, uncemented hemiarthroplasty advocates highlight the reduced operative time and absence of morbidity or mortality attributable to BCIS. Continued debate still exists regarding the optimal method of fixation of hemiarthroplasty in the setting of fracture. The hypothesis of this article was to determine if the use of a single brand of uncemented prosthesis in a single center was equivalent to published registry findings with respect to periprosthetic fractures and mortality.

This study was granted ethical approval by the Research Ethics Committee for the Galway University Hospitals Group. The authors sought to assess the rate of intraoperative and early postoperative deaths, and whether proximal femoral morphology and stem alignment were factors in intraoperative and postoperative PPF.

## Material and methods

This was a retrospective cohort study conducted at a tertiary orthopedic trauma center, in accordance with the Strengthening the Reporting of Observational Studies in Epidemiology guidelines. All uncemented hemiarthroplasties treated with an ODEP13A rated fully hydroxyapatite-coated titanium stem (Corail [DePuy], Warsaw, IN) from January 2008 to December 2018 were included. Both collared and noncollared Corail femoral prostheses were used. The first collared femoral prosthesis was used in 2013, with 12 collared prostheses used before 2015 and 142 after. The hospital has 10 consultant orthopedic surgeons and over 20 orthopedic trainees and treats 250 to 300 hip fractures annually. Care is delivered by either consultant orthopedic surgeons or trainees under supervision, which is reflective of many hospitals dealing with hip fractures. A decision to use a cemented or cementless femoral prosthesis was based on consultant preference, which was directed by fellowship training and experience. Consultants exclusively used cemented or cementless femoral prostheses and did not change between fixation methods. In 2018, 83% of arthroplasties for hip fractures in the authors’ hospital were uncemented [6]. Exclusion criteria consisted of patients who had an uncemented hemiarthroplasty using a different brand of stem, or patients who had total hip arthroplasty using an uncemented Corail (DePuy, J&J, Warsaw, IN) stem. After femoral neck fractures, patients were worked up and treated for osteoporosis in accordance with the national hip fracture guidelines.

Epidemiological data were collected by accessing the Irish national death register to determine date of death. Day-zero, 1-week, 30-day, and 1-year mortality were recorded. In addition, a final data point at the end of the study ascertained if the patient was still alive or not.

Radiographic analysis was conducted by 2 raters, specialist registrars in orthopedics with 6 and 7 years of training, respectively. Mean follow-up duration was recorded in years. If a patient did not have a follow-up radiograph on the hospital group system, their records were accessed on the national imaging system. This has 95% coverage for the entire country. The radiographs were analyzed for stem alignment, Dorr classification on preoperative radiographs [7], and gross undersizing [8]. This corresponded to a uniformly contiguous margin around the stem. Any radiographs that were not in agreement were analyzed by a third reviewer for consensus. Intraobserver correlation was conducted using a sample of 40% to test for internal consistency; with interobserver reliability assessed. The interobserver agreement was 0.76, with the intraobserver being 0.85.

Alignment was measured using the long axis of the stem and the long axis of the femur along an ordinal scale. A clinically significant intraoperative calcar fracture was deemed to be that which required intraoperative calcar cable fixation or went on to require cabling or revision in the early postoperative period.

### Statistical analysis

Statistical analysis was conducted using Minitab Statistical Software 17.0; Minitab, Inc., State College, PA. Fischer’s exact test was used to test for association for smaller numbers of PPF, and chi-square for larger numbers of PPF. Multiple logistic regression analysis was conducted to assess multiple parameters with one-way analysis of covariances per variable. A Kaplan-Meier survival graph was plotted for mortality.

## Results

Over the 11-year study period, 536 uncemented Corail (DePuy) hemiarthroplasties satisfied the inclusion criteria. The mean age was 80.4 years (standard deviation, 8.8; range, 44 to 102 years) of which 71.2% were female (Table 1). There was a mean radiographic follow-up of 433 days (0 to 3285 days). The breakdown of American Society of Anesthesiologists (ASA) grade was 34.5% for ASA grade 1 and 2 and 65.5% for ASA grade 3 and 4.

**Table 1:** Demographics of study population, femoral morphology, and prosthesis and outcomes.

Demographics	N	%
Total number of patients	536	
Male	154	28.7%
Female	382	71.3%
Mean age, y (standard deviation, range)	80.4	(8.8, 44-102)
ASA grade		
1	23	4.2%
2	162	30.2%
3	308	57.5%
4	43	8%
Dorr Classification		
Dorr A	86	16%
Dorr B	411	76.6%
Dorr C	39	7.3%
Femoral prosthesis		
Undersized	37	6.9%
Appropriately sized	499	93.1%
Collared femoral prosthesis		
Yes	154	28.7%
No	382	71.2%
Calcar fracture requiring treatment	20	3.7%
Intraoperative femur fracture	1	0.18%
Periprosthetic fracture postoperatively	14	2.6%
Further operations for all reasons (including PPF)	20	3.7%

### Mortality

**Figure 1:**
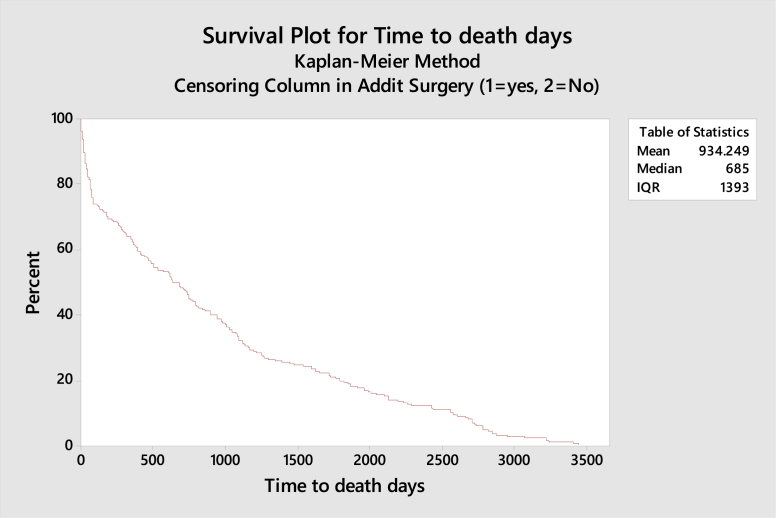
Kaplan-Meier curve for time to death.

Day-zero mortality for the cohort was 0%, 30-day mortality was 5.2%, and 1-year mortality was 17.5% (Table 2). Six patients (1.1%) died within 1 week. Overall mortality at study end date was 44.1% (Fig. 1). Males had a greater risk of mortality in this series (Fischer’s exact test, *P* = .02).

**Table 2:** Mortality rates per age group at study end.

Decades	Total no. of patients	Total no. of deaths	Deaths at 30 d	% Mortality 30 d	Deaths at 1 y	Cumulative mortality 1 y %	Deaths at >1 y	% Mortality by age group
50s	9	2	0	0	0	0	2	22.2
60s	55	16	1	1.8	7	16.4	8	29.1
70s	155	53	7	4.5	10	9.7	36	34.2
80s	241	120	16	6.6	31	14.6	77	49.7
90s	74	44	4	5.4	18	28.4	21	59.4
100s	2	1	0	0	1	0	1	50
	Death at 30 days: 5.2%				Death at 1 year: 17.5%

### Calcar fractures

There were 20 (3.7%) calcar fractures identified in this series that were fixed at the time of surgery (Table 3). There was no association between Dorr classification and the presence of a calcar fracture (chi square *P* = .765) or gross undersizing and calcar fractures (Fischer’s exact test *P* = .693). The presence of a calcar fracture was not significant for developing a PPF (*P* = .98) or any further surgery. Mortality was not increased by the presence of a calcar fracture (chi square, *P* = .10.)

**Table 3:** Dorr classification breakdown and fracture association.

Implant/Fracture	Dorr A (N)	Dorr A (%)	Dorr B (N)	Dorr B (%)	Dorr C (N)	Dorr C (%)	*P* value
Number	86		411		39		
Calcar fracture Yes	4	4.65	14	3.41	2	5.1	.765
Calcar Fracture No	82		397		37		
Collar Yes	44	51	100	24.3	9	23.1	<.001
Collar No	42		311		30		
Undersized Yes	4	4.65	23	5.6	10	25.6	.001
Undersized No	82		388		29		
PPF Yes	2	2.33	8	1.95	4	10.3	.049
PPF No	84		403		35		

### Dorr classification and associations

There was no statistical difference in Dorr groups and the presence of a calcar fracture (*P* = .76) (Table 3). There was a statistically significant difference between the Dorr classifications and presence of a PPF with 10.3% occurring in the Dorr C group, vs 1.9% in the Dorr B group and 2.3% in the Dorr A group (chi-square test of association, χ^2^ (1) = 6.03, *P* = .049). There was a statistically significant difference in gross undersizing between the groups (chi-square test, *P* = .001). The odds ratio (OR) was 5.6 times for the Dorr C group (CI, 1.7-16.3) to be undersized. The OR of the Dorr C group was 5.4 times that of the Dorr B group (CI, 1.7-16.3) and 2.4 times of the Dorr A (CI, 0.64-8.7). There was no statistically significant association between gross undersizing and calcar fractures (*P* = .693). There was no statistically significant association between sizing and PPF (Fischer’s exact test, *P* = .17) or all further reoperations (Fischer’s exact test, *P* = .97).

### Alignment and associations

The subtended angle between the long axis of the femur and the prosthesis was calculated, and the stem alignment was divided into valgus (>0 degree) and varus (<0 degree). Most femoral prostheses had a varus stem alignment (71.6%) (Table 4). There is no association between undersizing and alignment in this cohort, which was assessed using a regression analysis. There was no association with stems in varus and additional surgery for all reasons (N = 383) using a linear regression model (*P* = .852). There was no association with varus stems and PPF (*P* = .622). There was a positive association with valgus stems (N = 149) and additional surgery for all reasons, using a linear regression model (*P* = .025, r^2^ adjusted 2.69).

**Table 4:** Stem alignment.

Degrees	Valgus	Percentage of cohort	Varus	Percentage of cohort
0-2	128	23.8%	240	46.6%
2-5	20	3.7%	140	26.5%
>5	1	0.18%	4	0.75%
		27.68%		71.64%

Of those who sustained a PPF, 8 of 14 (57%) had valgus stem alignment vs 6 of 14 (42%) with varus stem alignment, which was significant (Fischer’s exact test, *P* = .029). There was an increasing association with valgus alignment and PPF (*P* = .032, r^2^ adjusted; 2.242)

### Sizing of implant and associations

The median size of the stem used in this cohort was 12 (Table 5). As the stem size increased, the risk of calcar fracture increased (*P* = .499; OR, 1.095; CI, 0.84-1.42). There was a trend between stem size and PPF (OR, 1.35; CI, 0.9954-1.83); however, this did not reach significance (*P* = .054).

**Table 5:** Stem sizes used in cohort.

Stem size	Calcar fractures	Number used
8	0	1
9	0	12
10	1	38
11	4	102
12	5	135
13	3	105
14	5	74
15	2	47
16	0	19
17	0	0
18	0	3
Total		536

### Collared vs noncollared stems

There were 4 calcar fractures in the collared group compared to 16 in the noncollared group. There was no difference in the presence/absence of collar with respect to PPFs (*P* = .512), additional surgeries (*P* = .357), or stem sizing (*P* = .192). Of the 14 PPFs, 4 occurred in collared stems and 10 in noncollared stems which was not statistically significant (*P* = .81) because of the small overall number of PPFs. The use of collared stems increased over the study period, with 12 collared stems used before 2015 and 142 after. Overall, 28.7% of patients received a collared stem.

### Further surgeries

The PPF rate for the cohort was 2.6% (N = 14), the dislocation rate was 0.9% (N = 5), and there was one intraoperative femur shaft fracture (0.018%). The mean time to PPF was 1.3 years (0 to 6.8 years). Of those who sustained a PPF, 2 patients underwent open reduction internal fixation, 6 patients revision to a diaphyseal bearing stem, and 6 patients a proximal femoral replacement. Only one stem in the PPF group was deemed to be grossly undersized. The 1-year mortality for those who suffered a PPF was 14.2% (n = 2), with a 1-year mortality for the entire study cohort of 17.5%. Of the 5 patients who dislocated, one femoral prosthesis was grossly undersized. Three of the 5 patients who dislocated required irrigation and debridement for deep infection. The 1-year mortality rate for all patients who underwent the revision surgery was 21.1% (n = 4).

## Discussion

Debate over the use of cemented or uncemented femoral prosthesis in the treatment of femoral neck fractures is ongoing. Recent studies have shown no difference in quality of life, pain, or EQ-5D-3L between cemented and uncemented groups preoperatively and at 1 year [9,10], while others have shown no difference in mortality at 1 year [2,9,10]. The guidance data for treatment of femoral neck fracture patients have been typically drawn from elective planned arthroplasty outcomes involving a relatively homogenous and robust cohort of patients. This cohort has very different demands and needs compared with the cohort of patients who sustain femoral neck fractures. Another significant factor is that studies and randomized controlled trials comparing uncemented stems to cemented stems in the treatment of proximal femur fractures involve relatively small number of patients [2,10,11]. The meta-analyses which exist on this topic also involved small numbers of uncemented stems. Veldman et al. [12] undertook a meta-analysis with 950 uncemented hemiarthoplasties; however, the largest single study of uncemented hemiarthroplasties included 108 cases. Complications related to BCIS, cardiovascular compromise, and mortality were acknowledged as underpowered in the meta-analysis. These are arguably the most important considerations when it comes to implant choice. Early and late mortality are 2 distinct entities and are often reported en bloc.

Mortality is one of the key outcomes used when comparing uncemented to cemented femoral stems. Mortality at day 0/1, 30 days, and 1 year after surgery was 0%, 5.2%, and 17.5%, respectively. Early mortality, described as mortality within day 0, day 1, or within 1 week, has been shown to be higher in cemented femoral stems. A large study examining the Norwegian hip fracture registry reported mortality of 0.5% at 0-1 days and 3% at 1 week for uncemented arthroplasties and 1% at day 0-1 and 3% at 1 week for cemented hemiarthroplasty [9]. A recent large single-institution study by Tan et al. of 751 cemented hemiarthroplasties demonstrated significantly higher early mortality at day zero of 5 deaths (0.67%) and 51 deaths at day thirty (6.8%) [13]. The present study has no day-zero or day-1 mortality, with a 30-day mortality of 5.2% only. Pripp et al. in a study of 11,210 patients found half of day one mortalities in the cemented hemiarthroplasty group could be associated with the use of cement [14]. A study by Grammatopoulos et al. [2] directly compared the 2 most common trauma implants Exeter (Stryker, Newbury, United Kingdom) (n = 292) and Corail (DePuy J&J, Warsaw, IN) (n = 120). They reported more intraoperative complications (fractures) in the uncemented group, but the cemented group again had a larger mortality in the early postoperative period. Rogmark et al. [15] demonstrated a subsequent PPF rate of 5.5% in uncemented stems in a large Swedish cohort, with this study demonstrating lower rates.

Since BCIS was defined in 2009 by Donaldson et al. [4], its incidence and impact are better understood and recognized. Olsen et al. [5] reviewed over 1000 cemented hemiarthroplasties for femoral neck fracture and found the total incidence of BCIS was 28%, with 1.7% suffering BCIS grade 3, severe cardiovascular collapse requiring cardiopulmonary resuscitation. The all-cause mortality at 30 days and 1 year in their cemented cohort was 9% and 29%, respectively, in comparison to 5.2% and 17.5% in this present study. Postoperative mortality is multifactorial, but a recurring theme highlighted by Olsen et al. [5] is the effect on intraoperative or early postoperative mortality of BCIS in a frail cohort. A study by Gjertsen et al. [16] comparing 8639 cemented implants to 2477 uncemented implants reported 26 on-table deaths with 15 cardiac arrests in the cemented group with only one death during surgery in the uncemented group. Although this increased rate of mortality has been shown to normalize and in some studies even favor cemented stems at 1 year, it is important that patients, families, and surgeons are aware of this increased risk when using cemented prosthesis in the femoral neck fracture cohort [12,17].

A concern with using uncemented femoral components is the risk of increased PPF [9]. The rate of PPF in the current cohort was 2.6% (N = 14) at a mean of 1.3 years after surgery (0-6.8 years). The 1-year mortality for these patients who have a PPF subsequent to their hemiarthroplasty was 14.2%; similar to findings from our unit for all femoral periprosthetic fractures, 12.4% [18]. Overall, these rates of PPFs are low, in keeping with other published studies for this prosthesis [19,20]. Patients with a Dorr type C proximal femur were more likely to sustain a PPF. This has not been shown before in a trauma cohort. Stems were more likely to be undersized and in valgus alignment in this group. Preoperative assessment of the DORR type should be performed as part of surgical planning. Surgeons should also consider the use of collared prosthesis if possible as both cohort and registry studies have shown reduced rates of PPF with such prostheses in total hip replacement [21,22].

The rate of calcar fracture in the present study was 3.7% (N = 20). However, calcar fractures did not have any impact on mortality, PPF, or all-cause revisions. This is often used as a direct comparator in the discussion between cemented and uncemented stem usage. As for total hip replacement with an uncemented stem, the surgeon must balance appropriate sizing of the femoral stem through compaction broaching to achieve press fit, without undersizing. Oversizing can result in a calcar fracture, especially in small women because of the narrow AP diameter of the femoral neck, but undersizing can also contribute to loosening [23]. Intraoperative awareness, and the use of a cable(s)/wire to treat identified calcar fractures, does not increase morbidity for the patient, nor affect their mobility status postoperatively [21]. There were 7 calcar fractures in the collared cohort compared to 13 in the uncollared cohort which was not significant (*P* = .512). No difference was seen in the rate of calcar fractures between these 2 groups; this possibly may be due to being underpowered. Collared prosthesis in total hip arthroplasty has been shown to reduce the rate of early PPF [22].

While this study is one of the largest studies examining mortality rates and outcomes for a single uncemented implant with follow-up, it is not without its limitations. The study is retrospective in nature. This limitation was offset by cross-referencing from multiple sources and follow-up using the Irish national death register to determine mortality. In order to ensure that radiographic measurements were reliable and accurate, a comprehensive interobserver and intraobserver reliability analysis was performed. The study only looks at one stem; it does not compare different uncemented stems being used in the unit. The Corail (DePuy) stem has been by far the most commonly used stem in the authors’ unit during the study period. Blood loss and length of hospital stay were not specifically looked at in this study. One could argue that excluding cemented cases, in particular for patients deemed at high risk for BCIS, introduces a bias and risks missing the cement-related day 0 or day 1 mortality. However, most surgeons in the unit favor uncemented stems for all hemiarthroplasty cases, obviating this potential for bias.

## Conclusions

Uncemented hemiarthroplasty can be used safely in the femoral neck fracture cohort. This study is the largest single-unit study with longitudinal follow-up, involving an ODEP13A uncemented femoral stem in a trauma setting. The present study has shown that uncemented stem usage in the setting of intracapsular hip fractures is associated with low reoperation rates and low perioperative, 30-day mortality rates in comparison to joint registries. Calcar fractures as long as recognized and cabled or managed appropriately intraoperatively have no clinical bearing on outcomes and should not be used in pooled analysis. Early mortality is perhaps being overlooked in the cemented hemiarthroplasty cohort, and greater caution should be used in those at risk of developing BCIS. This study informs practice with respect to alignment and proximal femur morphology which has not been studied before in the femoral neck fracture cohort. The results of this study demonstrate that using the Corail (DePuy) stem, an OPEP 13A-rated device, for femoral neck fractures is justified, providing satisfactory outcomes in the perioperative period, particularly for avoiding BCIS-related mortality, and with satisfactory long-term outcomes.

## Conflicts of interest

Author C. G. Murphy has received fees for educational events from J&J DePuy Synthes. No other authors have conflicts to declare.

## Appendix A Supplementary data

Conflict of Interest Statement for Cassar-Gheiti

Conflict of Interest Statement for Fenelon

Conflict of Interest Statement for Murphy

Conflict of Interest Statement for Murphy

Conflict of Interest Statement for O’Loughlin

Conflict of Interest Statement for Curtin
