# Supplementary material for: Does Proximal Femoral Morphology Impact Morbidity and Mortality? A Cohort Study of Uncemented Hemiarthroplasties in the Treatment of Femoral Neck Fractures
Source: Arthroplast Today. 2021 Jul 9;10:57–62. doi: 10.1016/j.artd.2021.06.010 (PMC8283011; doi:10.1016/j.artd.2021.06.010)
Supplement: Conflict of Interest Statement for Murphy [file mmc4.docx]

# CONFLICT OF INTEREST STATEMENT

***American Association of Hip and Knee Surgeons***

(Adopted from the American Academy of Orthopaedic Surgeons disclosure statement)

The following form **must be filled out completely and submitted by each author (example, 6 authors, 6 forms).**

**All items require a response. If there is no relevant disclosure for a given item, enter "*None*.”**

Manuscript Title: Does proximal femoral geometry impact morbidity and mortality? A cohort study of uncemented hemiarthroplasties in the treatment of femoral neck fractures.

1. Royalties from a company or supplier : None

2. Speakers bureau/paid presentations for a company or supplier None

3A. Paid employee for a company or supplier None

3B. Paid consultant for a company or supplier (None

3C. Unpaid consultants for a company or supplier None

4. Stock or stock options in a company or supplier None

5. Research support from a company or supplier as a Principal Investigator None

6. Other financial or material support from a company or supplier None

7. Royalties, financial or material support from publishers None

8. Medical/Orthopaedic publications editorial/governing board None

9. Board member/committee appointments for a society None

**Each author must sign AND print or type his/her name, date and submit a separate form**

In addition, one BLINDED Conflict of Interest form (no author names used) should be submitted per manuscript with all author disclosures.

Evelyn Murphy

EM 21/May/2021

Author Name (Print or Type) Author Signature Date
